# Canine Leptospirosis, United States, 2002–2004

**DOI:** 10.3201/eid1203.050809

**Published:** 2006-03

**Authors:** George E. Moore, Lynn F. Guptill, Nita W. Glickman, Richard J. Caldanaro, David Aucoin, Lawrence T. Glickman

**Affiliations:** * Purdue University, West Lafayette, Indiana, USA;; † VCA Antech, Los Angeles, California, USA

**Keywords:** surveillance, leptospirosis, serovars, canine, dispatch

## Abstract

The proportion of positive *Leptospira* microscopic agglutination tests for 23,005 dogs significantly increased from 2002 to 2004 (p<0.002) regardless of the positive cutoff titer used and was highest (p<0.05) for serovars Autumnalis and Grippotyphosa. The strongest positive serologic correlation (*r* = 0.72) was between serovars Autumnalis and Pomona.

Leptospirosis is a zoonotic disease with reservoirs in companion animals, livestock, and wild animals. More than 200 *Leptospira* serovars have been identified (*1*). Dogs are considered maintenance hosts for serovar Canicola, incidental hosts for other serovars, and are a potential source of infection for pet owners (*2*). Bivalent leptospirosis bacterins containing serovars Canicola and Icterohaemorrhagiae have been available for use in dogs since the 1960s. Despite use of these bacterins, canine leptospirosis diagnosed at US veterinary teaching hospitals has increased since 1990 (*3*). Case reports have attributed canine infection primarily to serovars Grippotyphosa, Pomona, Bratislava, and Autumnalis (*4**–**6*). Although human leptospirosis ceased to be a notifiable disease in 1994, outbreaks are still reported and infecting serovars in humans are antigenically related to the emerging serovars in dogs (*7**,**8*).

The referent method for serologic diagnosis of leptospirosis is the microscopic agglutination test (MAT). The serovar in which agglutination is detected at the highest dilution of serum is indicative of the infective serogroup, but cross-reactions between serovars is common (*9*). Laboratory databases are potentially useful for surveillance of zoonotic pathogens. Therefore, we determined the percentage of positive MAT results for leptospirosis for each seropositive state by using sera from dogs with suspected clinical leptospirosis that were submitted to a nationwide veterinary diagnostic laboratory. We also evaluated the statistical correlation of seropositivity between different serovars.

## The Study

The results of all leptospirosis MATs for dogs from January 2002 through December 2004 were obtained electronically from Antech Diagnostic Veterinary Laboratory (Los Angeles, CA, USA). Antech provides laboratory services to >18,000 US veterinary hospitals. The 7 *Leptospira* serovars included in the MATs were Canicola, Grippotyphosa, Icterohaemorrhagiae, Hardjo, Pomona, Autumnalis, and Bratislava. MAT results for each serovar were reported as the highest dilution of serum (1:100, 1:200, 1:400, 1:800, 1:1,600, 1:3,200, 1:6,400, or >1:12,800) at which >50% agglutination of organisms occurred when compared with a control suspension.

Calculation of seropositivity was performed separately by using cutoff titers of >400, >800, or >1,600. The percentage of seropositive test results for each serovar was calculated both as the number of positive test results divided by the total number of tests performed and by the total number of positive test results with 95% confidence limits. The percentage of seropositive MAT results was calculated by state and year. Proportions for categoric variables were compared by using the χ^2^ test for independence. A rank from 1 to 9 was assigned based on the serum dilution results. If 2 serovars had equivalent titers on a MAT for a dog, both serovars received the same rank score appropriate for that dilution. Correlation of seropositivity between all 2-way comparisons of serovars was by Spearman rank correlation. All calculations were performed by using SAS version 9.1.3 statistical software (SAS, Cary, NC, USA), and a p value >0.05 was considered significant. Tests that used paired sera from the same dog or tests repeated on the same dog at a different time could not be identified because patient identifiers were not included in the database. Therefore, a few individual dogs could have contributed >1 test to the dataset, but this possibility was considered uncommon.

During the study, 23,005 serum samples were submitted for a leptospirosis MAT, and ≈23,000 tests were performed for each of 5 serovars, namely Canicola, Grippotyphosa, Icterohaemorrhagiae, Hardjo, and Pomona (Table). Laboratory testing for serovars Autumnalis and Bratislava was initiated in 2003, and ≈11,600 tests were performed for each of these 2 serovars. The percentage of MATs that were positive significantly increased from 2002 to 2004 by using cutoff titers >400 (p<0.002), >800 (p<0.0001), or >1,600 (p<0.0001). At these 3 cutoff titers, the percentage of positive MAT results was greatest for serovars Autumnalis (9.1%, 6.5%, and 4.7%, respectively) and Grippotyphosa (6.4%, 4.9%, and 4.0%, respectively).

**Table:** Seropositivity for *Leptospira* serovars in dogs by the microscopic agglutination test using canine sera, 2002–2004*

Serovar	Total tests (n)	Positive test results					
		>400		>800		>1,600	
		n	% (95% CL)	n	% (95% CL)	n	% (95% CL)
Autumnalis	11,621	1,059	9.11 (8.60,9.65)	755	6.50 (6.06,6.96)	549	4.72 (4.35,5.13)
Grippotyphosa	22,929	1,458	6.36 (6.05,6.68)	1,132	4.94 (4.66,5.23)	908	3.96 (3.71,4.22)
Bratislava	11,663	499	4.28 (3.92,4.66)	428	3.67 (3.34,4.03)	357	3.06 (2.76,3.39)
Pomona	22,937	906	3.95 (3.70,4.21)	716	3.12 (2.90,3.35)	575	2.51 (2.31,2.72)
Canicola	22,377	669	2.99 (2.77,3.22)	317	1.42 (1.27, 1.58)	134	0.60 (0.50,0.71)
Icterohaemorrhagiae	22,935	356	1.55 (1.40,1.72)	179	0.78 (0.67, 0.90)	79	0.34 (0.27,0.43)
Hardjo	22,937	40	0.17 (0.12,0.24)	22	0.10 (0.06, 0.15)	10	0.04 (0.02,0.08)

*CL, confidence limit.

**Figure 1:**
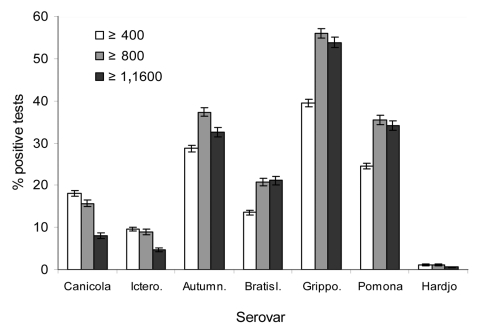
Percentage of positive microscopic agglutination tests by *Leptospira* serovar, using 3 different cutoff titers for 23,005 canine sera from 2002–2004. Serovars Canicola and Icterohaemorrhagiae have been used in canine bacterins for leptospirosis during the study period. Ictero., Icterohaemorrhagiae; Autumn., Autumnalis; Bratisl., Bratislava; Grippo., Grippotyphosa.

**Figure 2:**
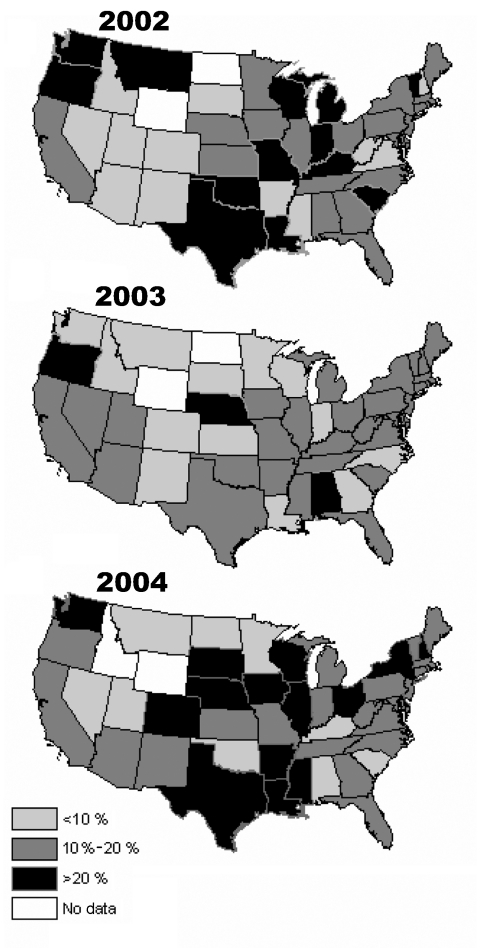
Canine leptospirosis microscopic agglutination test results shown as the percentage positive and standard error, by state and year from 2002–2004. A test was considered positive if the titer for any serovar was >400 for Autumnalis, Bratislava, Canicola, Grippotyphosa, Icterohaemorrhagiae, Pomona, or Hardjo serovars.

The proportion of positive MAT results attributable to serovars Canicola or Icterohaemorrhagiae declined as the cutoff titer increased, while it generally increased for serovars Autumnalis, Bratislava, Grippotyphosa, and Pomona (Figure 1). No consistent or distinct geographic pattern for positive MAT results was observed in the study (Figure 2), but seropositivity was greater in the midwest, south-central, and northwest regions of the United States.

Moderately strong positive correlation in seropositivity (*r*, 0.59–0.72) was present between serovars Autumnalis, Pomona, Grippotyphosa, and Bratislava, with the strongest correlation between serovars Autumnalis and Pomona. In contrast, weak positive correlation (*r* = 0.36) was found between serovars Canicola and Icterohaemorrhagiae, and each of these serovars was weakly correlated (*r*, 0.20–0.33) with serovars Autumnalis, Pomona, Grippotyphosa, and Bratislava. All rank correlation coefficients were significant at p<0.0001. Serovar Hardjo was excluded from correlation analysis because of the small number of positive test results.

## Conclusions

Positive leptospirosis MAT results in dogs may indicate natural infection due to direct or indirect contact with wildlife maintenance hosts or recent vaccination (*2*). However, titers >800 from vaccination are considered unlikely as postvaccinal titers wane rapidly (*10*) and most leptospiral bacterins available for dogs are bivalent for Canicola and Icterohaemorrhagiae, 2 serovars with low seropositivity in this study. Although the health and vaccination status of dogs from which sera were submitted was unknown, veterinarians most likely submitted samples for leptospirosis testing when they suspected leptospirosis based on clinical signs including vomiting, fever, lethargy, and anorexia.

The most common serovar associated with a positive MAT result was Autumnalis, a serovar not currently included in licensed canine bacterins. Reactivity to this serovar in the MAT has been considered a possible paradoxical cross-reaction with serovar Pomona (*11*); a strong positive correlation in titers for these 2 serovars was found in this study. The Autumnalis serovar has been isolated from raccoons in the southern United States (*12*), and seropositivity in dogs may represent natural infection from this source. The MAT is not serovar-specific, but the 7 serovars evaluated in this study belong to different serogroups (*13*). Serovar Grippotyphosa, the second most common positive serovar in this study, has also been associated with human leptospirosis outbreaks in the 1990s (*8*).

The finding of a moderately high correlation in serologic reactivity between serovars Autumnalis, Pomona, Grippotyphosa, and Bratislava suggests that cross-protection to Autumnalis could be induced by current bacterins that lack this antigen. Canine vaccines are now available with serovars Grippotyphosa and Pomona as well as the traditional serovars Canicola and Icterohaemorrhagiae. This vaccine may confer some immunity to serovar Autumnalis, since some protein antigens are highly conserved among several pathogenic serovars (*14*).

Limitations of the present study included the inability to determine if multiple tests had been performed for individual dogs, lack of data on clinical signs, and unknown vaccination status of the dogs. The geographic distribution of serologic reactivity during the study, however, indicates broad dispersion of *Leptospira* pathogens that pose a risk to both domestic animals and humans.

Dogs in suburban or rural environments have been shown to be at increased risk of leptospirosis (*15*), presumably because of greater likelihood of contact with wildlife habitats. Dogs may be sentinels for human exposure to this zoonotic organism. Veterinary practitioners and public health officials need to be aware of the potential change in the ecologic environment and circulating endemic strains for this zoonotic organism.
